# Metabolic fingerprinting of *Arabidopsis thaliana* accessions

**DOI:** 10.3389/fpls.2015.00365

**Published:** 2015-05-27

**Authors:** Mariana Sotelo-Silveira, Anne-Laure Chauvin, Nayelli Marsch-Martínez, Robert Winkler, Stefan de Folter

**Affiliations:** ^1^Unidad de Genómica Avanzada (LANGEBIO), Centro de Investigación y de Estudios Avanzados del Instituto Politécnico Nacional (CINVESTAV-IPN)Irapuato, México; ^2^Laboratorio de Bioquímica, Departamento de Biología Vegetal, Facultad de Agronomía, Universidad de la RepúblicaMontevideo, Uruguay; ^3^Department of Biotechnology and Biochemistry, CINVESTAV Unidad IrapuatoIrapuato, Mexico

**Keywords:** metabolic phenotyping, Arabidopsis, accessions, development, metabolites

## Abstract

In the post-genomic era much effort has been put on the discovery of gene function using functional genomics. Despite the advances achieved by these technologies in the understanding of gene function at the genomic and proteomic level, there is still a big genotype-phenotype gap. Metabolic profiling has been used to analyze organisms that have already been characterized genetically. However, there is a small number of studies comparing the metabolic profile of different tissues of distinct accessions. Here, we report the detection of over 14,000 and 17,000 features in inflorescences and leaves, respectively, in two widely used *Arabidopsis thaliana* accessions. A predictive Random Forest Model was developed, which was able to reliably classify tissue type and accession of samples based on LC-MS profile. Thereby we demonstrate that the morphological differences among *A. thaliana* accessions are reflected also as distinct metabolic phenotypes within leaves and inflorescences.

## Introduction

Biodiversity constitutes a valuable resource for searching genes of interest. Natural variation in Arabidopsis has been observed for a variety of traits (Koornneef et al., 2004; Weigel, 2012) like seed size (Alonso-Blanco et al., 1999), light and hormone sensitivity (Maloof et al., 2001), growth rate (Beemster et al., 2002), root growth responses to phosphate starvation (Chevalier et al., 2003), and cold stress responses (Barah et al., 2013), among others. Comparison of whole genomes from *Arabidopsis thaliana* accessions showed that genetic differences exist among them, for instance, over 200 genes found in different accessions are not present in the reference genome Col-0 (Gan et al., 2011; Schneeberger et al., 2011). Furthermore, natural variation has also been studied at the transcriptomic (Gan et al., 2011; Stein and Waters, 2012; Wang et al., 2013) and proteomic (Chevalier et al., 2004) levels.

Metabolomics is adding another dimension to investigate gene function (Fiehn et al., 2000; Saito and Matsuda, 2010). Metabolic analysis methods such as profiling and fingerprinting have evolved from diagnostic tools used to elucidate metabolite accumulation patterns in different tissues and cell compartments of individual plants (Matsuda et al., 2009, 2010, 2011; Krueger et al., 2011; Mintz-Oron et al., 2012) to integrative tools, enhancing the strength of functional genomics in the process of shortening the distance of the genotype-phenotype gap (Fiehn et al., 2000; Taylor et al., 2002; Enot and Draper, 2007; Fernie and Schauer, 2009; García-Flores et al., 2012, 2015; Landesfeind et al., 2014). Recently, the attention in this area has expanded to the study of natural variation of metabolite levels between individual plants, a strategy that is suggested to provide useful information to improve crop quality (Fernie and Schauer, 2009; Montero-Vargas et al., 2013). In this sense, several studies in Arabidopsis combining metabolomic and QTL analysis showed that metabolite variation between different accessions exists (Keurentjes et al., 2006, 2008; Rowe et al., 2008; Fu et al., 2009; Chan et al., 2010; Joseph et al., 2013, 2014), and highlighted that interactions between transcript and metabolite variation are complex and governed by epistatic interactions (Wentzell et al., 2007; Rowe et al., 2008; Joseph et al., 2013, 2014). Moreover, the metabolic relationship between accessions depends on different factors like tissue, plant age, and environment (Wentzell et al., 2008; Wentzell and Kliebenstein, 2008; Houshyani et al., 2012).

In the present work, we present a metabolite profiling study of *A. thaliana* accessions frequently used in the laboratory: Columbia (Col-0) and Wassilewskija (Ws-3) (Alonso-Blanco and Koornneef, 2000). Col-0 was selected from the original Laibach Landsberg population and is the accession that was sequenced in the Arabidopsis Genome Initiative (Rédei, 1992; AGI, 2000), and Ws-3 is a Russian accession (Laibach, 1951).

We investigated whether a distinct metabolic phenotype in two different tissues could be distinguished besides the morphological and developmental differences observed among the Arabidopsis accessions.

## Material and methods

### Plant growth and plant material

Col-0 and Ws-3 accessions of Arabidopsis (*A. thaliana*) plants were germinated in soil (3:1:1, peat moss:perlite:vermiculite) in a growth chamber at 22°C under long-day conditions (16 h of light/8 h of dark) and transferred to standard greenhouse conditions (22–27°C, natural light). All plants were grown at the same time under the same environmental conditions.

### Sample preparation

Fully expanded leaves after flowering, and inflorescences from 10 plants were collected and pooled. Each pool was collected from different plants. Three biological replicas were used for each accession (with exception of Ws-3 leaves with only two biological replicas). Frozen plant material (fully expanded leaves after flowering or whole flowers) was ground in liquid nitrogen. For each 100 mg of fresh tissue, 300 μL of cold acetone was added, and the mixture was vortexed, sonicated for 5 min, and then centrifuged at 16,100 g to separate the crude extract from the tissue, as previously described (Sotelo-Silveira et al., 2013). The supernatant was lyophilized and used for analysis. The lyophilized samples were dissolved in 1000 μL of 100% MeOH and filtered through a 0.22 μm filter before the injection into the chromatographic column. We used for each biological replica two analytical replicas, giving in total 12 inflorescence and 10 leaf samples that were injected (SQLite database; Supplemental Data 3).

### Chromatography

Chromatographic separation was performed on a ACQUITY BEH C-18 column (2.1 × 50 mm i.d., 1.7 μm, Waters, Mexico) using an ACQUITY UPLC system (Waters Corps., Mexico), as previously described (Sotelo-Silveira et al., 2013). The column was maintained at 35°C and eluted with a 30 min gradient. The mobile phase, at a flow rate of 0.2 mL/min, consisted of a starting mixture of solvents A: B (MeOH: H_2_O; 1: 9; A: 100% MeOH; and B: H_2_O + 0.1% formic acid). A decrease of solvent B up to 20% over 15 min was then performed. Solvent B was returned to its initial composition over 1 min and the initial condition was maintained for 15 min in order to equilibrate the column. The volume of sample injected onto the column was 5 μL.

### Mass spectrometry

The eluent was introduced into the Q-Tof mass spectrometer (LCT Premier™ XE, Waters Corps. Mexico) by electrospray ionization, with capillary and cone voltages set in the positive ion mode to 3100 and 70 V, as previously described (Sotelo-Silveira et al., 2013). The desolvation gas was set to 850 L/h at a temperature of 350°C for the positive mode. The cone gas was set to 10 L/h, and the source temperature to 80°C for the positive mode. Continuum data were acquired from m/z 50–1000 using an accumulation time of 0.2 s per spectrum. All spectra were mass corrected in real-time by reference to leucine enkephalin (2 μg/mL), infused at 5 μL/min through an independent reference electrospray. The resolution of the system was 11,000 for the positive mode.

### Data analysis

Waters LCT Premier™ XE ^*^.*raw* data files were converted to ^*^.*mzML* community standard data format using the ProteoWizard (Chambers et al., 2012) and processed with an OpenMS/TOPPAS pipeline (Sturm et al., 2008). A TOPPAS workflow containing the detailed parameters is provided as Supplemental Material (Supplemental Data 1). In short, the LC-MS features of each data set were detected with the FeatureFinderMetabo tool and subsequently merged to create a consensus map. The consensus features were exported to plain text format and manually analyzed using standard text processing and spreadsheet programs.

Only high-quality (HQ) features, which were quantified in all evaluated 12 inflorescence or all 10 leaf samples, respectively, were used for further data analyses. In total 803 such HQ features were found for the inflorescence samples and 561 for the leaf samples. For identifying the HQ features, a metabolite database (DB) for Arabidopsis was created from the KNApSAcK database (http://kanaya.naist.jp/knapsack_jsp/top.html) (Afendi et al., 2012) and experimental liquid-chromatograph mass spectrometry (LC-MS) literature data. Automated DB generation and MS data matching were performed using SpiderMass (Winkler, 2015). The SpiderMass Meta-DB for Arabidopsis is provided as Supplemental Data 2. Mass spectrometry data processing was performed on the analysis platform MASSyPup (Winkler, 2014).

Consensus features, HQ features and putative metabolite identifications with their compound classes were integrated into a SQLite (https://sqlite.org/) database, which is available as Supplemental Data 3. For statistical analysis we used the R script “MetabR” (Ernest et al., 2012), which calculates the fold-changes and *p*-values according to Tukey's Honest Significant Difference (HSD).

The R package and Graphical User Interface (GUI) “Rattle” (Williams, 2009, 2011) was employed to create and evaluate classification models for the metabolic data sets. Due to the characteristics of the data, i.e., relatively few samples, but multiple numeric variables, we decided for a Random Forest model (Williams, 1987, 1988) (**Figure 3**). For the model training sets, we only considered features present in all 22 LC-MS datasets. To avoid the necessity of imputing values, all features with missing data were omitted. We created a meta-variable “Accession_Tissue,” which describes the four possible combinations of our experiment and which was used as the target variable. In total, 16 datasets with 460 metabolic input variables were used for the model building. 10,000 decision trees were calculated. The number of selected variables was set to the square root of the number of variables (suggested default), which corresponded to 21 variables. The evaluation of the models was done with five testing datasets that represented the four possible combinations of tissue type and accession. The Rattle project, which contains the final model and the metabolic feature data, is provided as Supplemental Data 4.

## Results

### Selection of accessions

**Figure 1:**
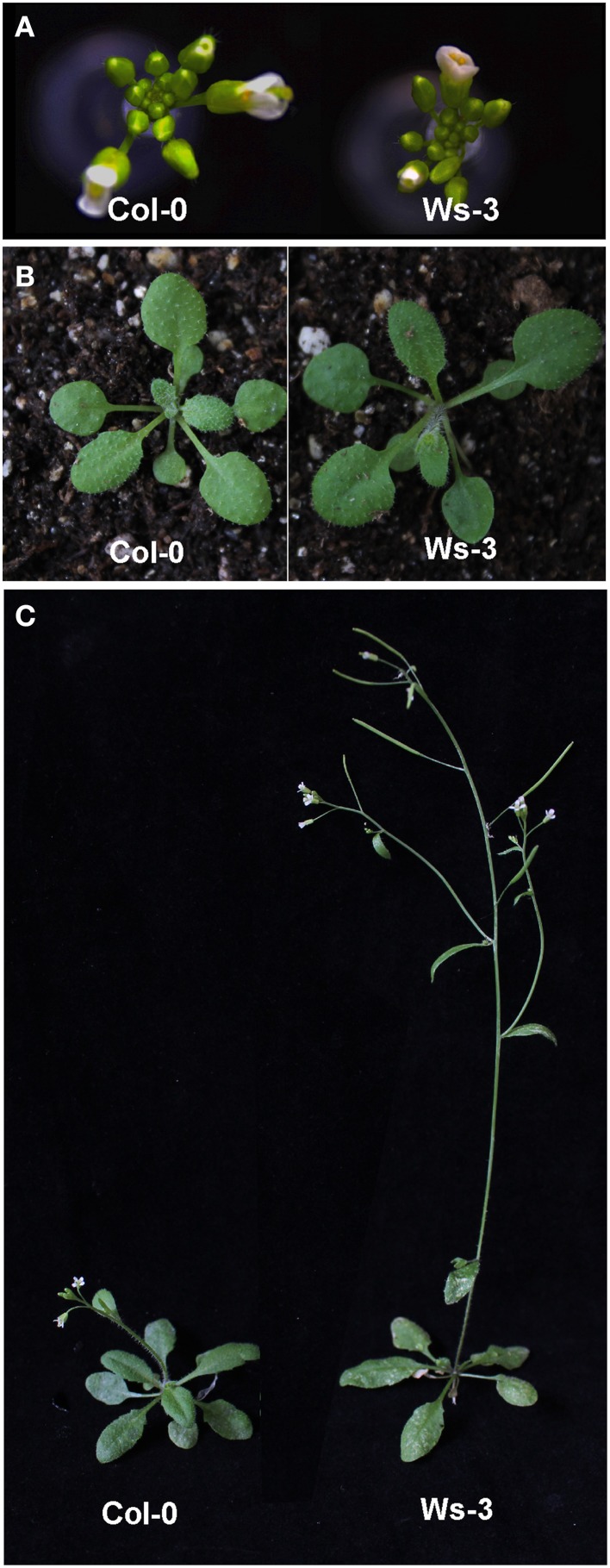
**Phenotypes of the** ***Arabidopsis thaliana*** **accessions used. (A)** View of the inflorescence of Columbia (Col-0) and Wassilewskija (Ws-3). **(B)** View of the rosette before flowering of Columbia (Col-0) and Wassilewskija (Ws-3). **(C)** General architecture of Columbia (Col-0) and Wassilewskija (Ws-3) plants when flowering. In **(A, C)**, photographs were taken 3 weeks after sowing. Plants from the different accessions were cultivated at the same time under greenhouse conditions.

*A. thaliana* has over 1000 natural accessions that have been collected from around the world (Alonso-Blanco and Koornneef, 2000; Gaut, 2012; Horton et al., 2012). Natural accessions are very variable in terms of shape, development, and physiology (Weigel, 2012). Plants of the commonly used laboratory strains (or accessions), Columbia (Col-0) and Wassilewskija (Ws-3), are distinguishable based on their morphology and development (Figure 1). Particularly, they show differences in rosette leaf development and flowering time. Col-0 plants produce more rosette leaves, have a longer duration of the leaf production period (i.e., they flower later), and have a final rosette leaf area significantly larger than Ws-3 (Massonnet et al., 2010) (Figure 1).

### Distinct metabolic phenotypes were detected for each accession and tissue

To assess the natural variation in metabolite content among Arabidopsis accessions in two different tissues, we performed UPLC-QTOF MS-based untargeted metabolic fingerprinting of crude acetone extracts from leaves and inflorescences collected and pooled from Col-0 and Ws-3. Notably, using an organic solvent favors an extraction toward hydrophobic compounds, which are under-represented in studies using polar solvent mixtures.

**Figure 2:**
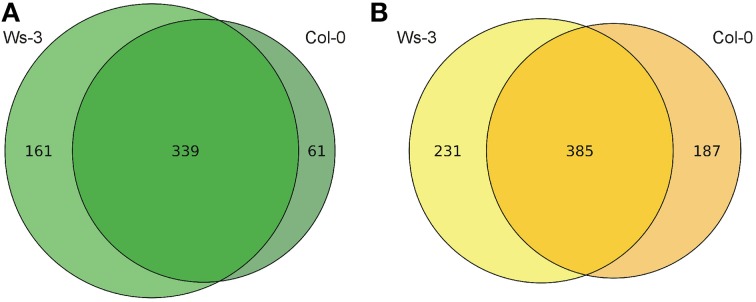
**Venn Diagrams of the high quality metabolic features. (A)** Features with increased abundance in the leaf samples (Ws-3 vs. Col-0). **(B)** Features with increased abundance in the inflorescence samples (Ws-3 vs. Col-0).

The metabolic profiles demonstrate considerable quantitative and qualitative differences between the tissues and accessions. More than 14,000 and 17,000 features from inflorescences and leaves, respectively, were detected in the two accessions (SQLite database; Supplemental Data 3). In total 803 high quality features from inflorescences and 561 from leaves, which were quantified in all evaluated 12 inflorescence or all 10 leaf samples, respectively, were considered for identification through searching in databases of metabolites (Supplemental Data 2). In leaf samples, 222 high quality features presented significant differences (*p* ≤ 0.01) and in inflorescences samples 418 high quality features (Figure 2). From these metabolites that presented significant differences we could putatively identify 26 and 36 metabolites in leaf and in inflorescence samples, respectively (Tables 1, 2).

**Table 1:** **Relative amounts of metabolites putatively identified by ACQUITY UPLC-LCT Premier XE (Waters) in crude extracts from leaves of Ws-3 and Col-0** ***Arabidopsis thaliana*** **accessions**.

**Class**	**m/z**	**Ionization mode**	**Name**	**Chem Spider ID**	**Fold change (Ws-3/Col-0)**	***p*-value**
1	195.0648	[M+H]+	Ferulic acid	393368	2.4	7.85E-003
1	197.0803	[M+H]+	5-Hydroxyconiferyl alcohol	4445309	1.4	2.52E-002
1	311.1692	[M+H]+	Sinapine	80576	0.7	2.93E-005
1	363.0737	[M+Na]+	Sinapoyl-(S)-malate	4444177	0.9	7.82E-002
2	273.1457	[M+Na]+	5-(4-Hydroxy-2,2,6-trimethyl-7-oxabicyclo[4.1.0]hept-1-yl)-3-methyl-2,4-pentadienal	21172770	1.4	1.79E-003
2	333.1742	[M+H]+	Gibberellin A4	10222155	2.0	1.36E-002
2	343.2645	[M+Na]+	1,22-Docosane diol	190585	1.5	1.79E-005
2	355.1444	[M+Na]+	Gibberellin A51	391672	1.4	3.40E-002
2	355.1579	[M+Na]+	Gibberellin A20	20015789	0.6	3.72E-003
2	369.1222	[M+Na]+	Gibberellic acid	6223	1.3	6.84E-003
3	311.0458	[M+Na]+	(+)-Dihydrokaempferol	109514	2.3	1.03E-006
3	323.0307	[M+H]+	Cyanidin 3-O-[2″-O-(2″′-O-(sinapoyl) xylosyl) glucoside] 5-O-glucoside	61546	3.3	2.54E-005
3	329.0675	[M+Na]+	Leucocyanidin	64694	1.5	8.34E-003
3	595.1585	[M+H]+	Kaempferol-3-O-beta-D-galactoside-7-O-alpha-L-rhamnoside	28481780	0.7	5.40E-002
4	223.1695	[M+Na]+	Lauric acid	3756	0.4	4.25E-004
5	190.0039	[M+Na]+	Quinolinic acid	1037	0.6	2.57E-006
5	199.0751	[M+Na]+	N-hydroxy tryptamine	10391819	1.7	2.50E-004
6	223.0572	[M+Na]+	5-Methylsufinylpentyl nitrile	1363309	0.8	5.64E-002
6	235.0595	[M+H]+	2-(4′-Methylthio)butylmalic acid	24784695	2.0	5.15E-005
6	256.1438	[M+Na]+	Hexahomomethionine	21865788	0.7	1.22E-003
7	221.0315	[M+H]+	3-(1H-Imidazol-4-yl)-2-oxopropyl dihydrogen phosphate	770	1.1	1.94E-003
7	244.0489	[M+Na]+	L-Histidinol phosphate	388515	1.2	6.71E-003
7	275.1138	[M+Na]+	D-Coenzyme A	2654	1.4	7.87E-003
7	313.0989	[M+Na]+	N-Succinyl-LL-2,6-diaminoheptanedioate	1160	3.1	1.36E-011
8	343.1212	[M+H]+	Sucrose	5768	0.9	2.74E-002
9	250.0505	[M+H]+	[5-Hydroxy-4-(hydroxymethyl)-6-methyl-3-pyridinyl]methyl dihydrogen phosphate	1026	1.3	3.72E-003

*These metabolites were differentially accumulated among accessions and tissues*.

**Table 2:** **Relative amounts of metabolites putatively identified by ACQUITY UPLC-LCT Premier XE (Waters) in crude extracts from inflorescences of Ws-3 and Col-0** ***Arabidopsis thaliana*** **accessions**.

**Class**	**m/z**	**Ionization mode**	**Name**	**Chem Spider ID**	**Fold change (Ws-3/Col-0)**	***p*-value**
1	211.0570	[M+H]+	5-Hydroxyferulic acid	141117	0.6	1.28E-003
1	211.0931	[M+H]+	Sinapyl alcohol	4444145	1.5	1.60E-009
1	249.0287	[M+Na]+	Isochorismate	787	1.2	5.05E-007
1	365.1282	[M+Na]+	Coniferin	4444067	0.7	9.54E-004
2	251.0213	[M+Na]+	Mevalonate 5-phosphate	463	1.3	1.86E-005
2	265.1432	[M+H]+	(s)-(+)-Abscisic acid	4444418	1.3	2.69E-003
2	271.1319	[M+Na]+	Abscisic acid aldehyde	14797236	1.1	2.00E-003
2	273.1459	[M+Na]+	5-(4-Hydroxy-2,2,6-trimethyl-7-oxabicyclo[4.1.0]hept-1-yl)-3-methyl-2,4-pentadienal	21172770	1.3	2.87E-006
2	287.1312	[M+Na]+	(s)-(+)-Abscisic acid	4444418	1.7	1.79E-004
2	331.1579	[M+H]+	Gibberellin A5	4445248	1.2	7.78E-003
2	333.1739	[M+H]+	Gibberellin A51	391672	1.3	9.66E-003
2	343.2640	[M+Na]+	1,22-Docosane diol	190585	1.8	6.86E-007
2	514.2115	[M+H]+	Zeatin riboside-O-glucoside	9887971	0.9	8.68E-003
3	323.0319	[M+H]+	Cyanidin 3-O-[2″-O-(xylosyl) glucoside] 5-O-(6″′-O-malonyl) glucoside	61546	2.3	1.02E-008
3	329.0680	[M+Na]+	Leucocyanidin	64694	1.2	1.20E-004
3	595.1589	[M+H]+	Quercetin-3,7-O-a-L-dirhamnopyranoside	13095553	3.6	1.02E-008
3	611.1567	[M+H]+	Quercetin-3-O-b-glucopyranosyl-7-O-a-rhamnopyranoside	29273176	2.3	3.94E-009
4	209.1537	[M+Na]+	Undecanoic acid	7888	0.8	5.84E-005
4	223.1694	[M+Na]+	Lauric acid	3756	1.3	3.37E-004
4	233.1163	[M+Na]+	(+)-Epijasmonic acid	5584839	1.2	1.72E-002
4	249.1849	[M+Na]+	Myristoleic acid	4444564	1.4	2.82E-004
4	825.4698	[M+Na]+	Arabidopside B	10192487	0.8	5.02E-002
5	363.0392	[M+H]+	Xanthosine 5′-monophosphate	10463791	2.0	8.22E-008
6	223.0570	[M+Na]+	5-Methylsufinylpentyl nitrile	1363309	0.6	2.13E-004
6	256.1437	[M+Na]+	Hexahomomethionine	21865788	0.6	1.65E-002
6	465.0834	[M+H]+	6-Methylsulfinylhexyl glucosinolate	24784958	2.5	5.92E-009
7	188.0738	[M+Na]+	Phenylalanine	969	3.6	8.82E-005
7	199.0751	[M+Na]+	N-hydroxy tryptamine	10391819	1.3	1.84E-003
7	213.0917	[M+Na]+	Meso-2,6-Diaminoheptanedioate	89700	1.7	2.94E-005
7	221.0315	[M+H]+	Imidazole acetol phosphate	770	1.1	4.32E-005
7	231.0831	[M+Na]+	L-Kynurenine	141580	0.8	3.71E-002
7	244.0489	[M+Na]+	L-Histidinol phosphate	388515	1.1	5.83E-004
7	385.1300	[M+H]+	2-Amino-4-({[5-(6-amino-9H-purin-9-yl)-3,4-dihydroxytetrahydro-2-furanyl]methyl}sulfanyl)butanoic acid	188	1.3	2.60E-004
8	328.9407	[M+Na]+	D-Ribulose 1,5-bisphosphate	19951541	0.6	1.48E-007
8	365.1075	[M+Na]+	Sucrose	5768	1.7	8.78E-005

*These metabolites were differentially accumulated among accessions and tissues*.

**Figure 3:**
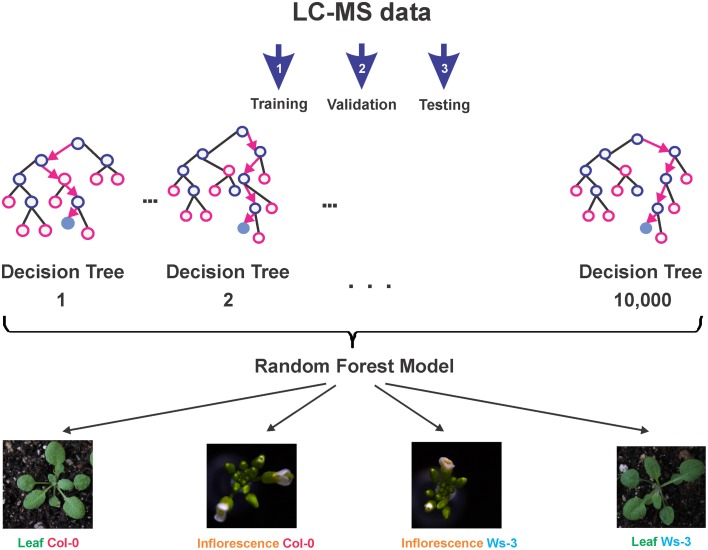
**Overview of the predictive Random Forest Model development**. First the model is trained (Step 1), then the model is validated (Step 2), and finally the model is tested (Step 3). The final model (Supplemental Data 4) is able to reliably classify tissue type and accession, based on the LC-MS profile of a sample.

To evaluate the possibility, to identify tissue types and their accessions based on their metabolic profiles, we created a predictive Random Forest Model (Figure 3). During the training of the model, an error rate of 12.5% was estimated. Applying the final model to a testing dataset (which was not involved in the model building) resulted in the Error Matrix shown in Table 3. All five test samples were identified correctly.

**Table 3:** **An Error Matrix demonstrates the performance of the Random Forest Model for the correct classification of tissue type and accession of Arabidopsis thaliana**.

	**Predicted**			
**Actual**	**Col-0_Inflor**	**Col-0_Leaf**	**Ws-3_Inflor**	**Ws-3_Leaf**
Col-0_Inflor	**1**	0	0	0
Col-0_Leaf	0	**2**	0	0
Ws-3_Inflor	0	0	**1**	0
Ws-3_Leaf	0	0	0	**1**

*Inflor: Inflorescence*.

*The bold values indicate that the actual identities of the samples are equal to the predictions*.

Consequently, the metabolic identity of both, tissues and accessions, is sufficiently distinct to allow for a reliable classification with a Random Forest Model using LC-MS data.

### Metabolites differentially accumulated among accessions and tissues

To better understand the variation among tissues of the different accessions we focused in the analysis on putative identified metabolites with significant differences that belong to one of the KEGG pathways (Kanehisa et al., 2014) of *A. thaliana*. With this criterion putatively identified metabolites were classified into 9 classes (Tables 1, 2), and each class was analyzed to identify whether a conserved accumulation pattern among samples and accessions was present. We also searched for changes in the presence or difference in accumulation of specific metabolites in the different tissues and/or accessions, and these are described below for each class when pertinent.

#### Class 1: phenylpropanoids, monolignols, and sinapate derivatives

Four metabolites were found in leaf and inflorescence samples that were classified as belonging to class 1 (Tables 1, 2). Interestingly, qualitative and quantitative differences were found among tissues (Tables 1, 2). Furthermore, each accession has different abundance of metabolites reflected by the fold change in the intensity of each m/z. Col-0 leaves accumulated more m/z 311.1692 and 363.0737, putatively identified as Sinapine and Sinapoyl-(S)-malate, respectively, whereas in Ws-3 leaf samples more m/z 195.0648 and 197.0803, putatively identified as Ferulic acid and 5-Hydroxyconiferyl alcohol, respectively (Table 1).

Inflorescences from Col-0 accumulated more of m/z 211.0570 and 365.1282, putatively identified as 5-Hydroxyferulic acid and Coniferin, respectively, whereas Ws-3 inflorescences accumulated more m/z 249.0287 and 211.0931, putatively identified as Isochorismate and Sinapyl alcohol, respectively (Table 2).

#### Class 2: prenol lipids, terpenoid backbone biosynthesis mevalonate and MEP/DOXP pathways

Six and nine metabolites found in leaf and inflorescence samples belong to class 2. Interestingly, qualitative differences were identified among tissues, like m/z 369.1222 that was present in leaf (Table 1), but not in inflorescence samples (Table 2). On the contrary, m/z 251.0213, 265.1432, 287.1312, and 514.2115 were detected in inflorescence, but not in leaf samples. Some of the metabolites were putatively identified as known hormones or as hormone precursors (Tables 1, 2).

In leaf samples of Col-0, the more abundant metabolite was m/z 355.1579, putatively identified as Gibberellin A20 (Table 1). The other compounds detected were accumulated more in Ws-3 leaf samples, m/z 273.1457, 333.1742, 343.2645, 355.1444, and 369.1222, which were identified as 5-(4-Hydroxy-2,2,6-trimethyl-7-oxabicyclo[4.1.0]hept-1-yl)-3-methyl-2,4-pentadienal, Gibberellin A4, 1,22-Docosane diol, Gibberellin A51, and Gibberellic acid, respectively (Table 1).

On the other hand, Col-0 inflorescence samples showed more abundance of m/z 514.2115, putatively identified as Zeatin riboside-O-glucoside, whereas Ws-3 inflorescence samples showed more accumulation of m/z 251.0213 (Mevalonate 5-phosphate), 265.1432 [(s)-(+)-Abscisic acid], 271.1319 (Abscisic acid aldehyde), 287.1312 [(s)-(+)-Abscisic acid], 331.1579 (Gibberellin A5), 333.1739 (Gibberellin A51), 343.2640 (1, 22-Docosane diol), and 354.1815 (Dihydrozeatin riboside) (Table 2).

#### Class 3: biosynthesis of phenylpropanoids, biosynthesis of flavonoids, flavonones

Four metabolites detected in leaf and four in inflorescence samples belong to class 3. They were putatively identified as phenylpropanoids (Flavonols, Flavonones, Flavones, Anthocyanines, and Leucoanthocyanidins). Leaf and inflorescence samples showed distinctive metabolites, like m/z 311.0458 and 595.1585 that were present only in leaves (Table 1), and m/z 595.1589 and 611.1567 that were detected only in inflorescences (Table 2).

Leaves from Col-0 showed more accumulation of m/z 595.1585, putatively identified as Kaempferol-3-O-beta-D-galactoside-7-O-alpha-L-rhamnoside, while Ws-3 leaves accumulated more m/z 311.0458, 323.0307, and 329.0675, putatively identified as (+)-Dihydrokaempferol, Cyanidin 3-O-[2″-O-(2″′-O-(sinapoyl) xylosyl) glucoside] 5-O-glucoside, and Leucocyanidin, respectively (Table 1).

In contrast, Ws-3 inflorescence samples accumulated more m/z 323.0319, 329.0680, 595.1589, and 611.1567, putatively identified as Cyanidin 3-O-[2″-O-(xylosyl) glucoside] 5-O-(6″′-O-malonyl) glucoside, Leucocyanidin, Quercetin-3,7-O-a-L-dirhamnopyranoside, and Quercetin-3-O-b-glucopyranosyl-7-O-a-rhamnopyranoside, respectively.

#### Class 4: fatty acids, fatty acyls, octadecanoids, jasmonic acid

One and five metabolites were found in leaf and inflorescence samples, respectively, that belong to class 4.

Col-0 leaf samples have more m/z 223.1695, putatively identified as Lauric acid (Table 1).

Col-0 inflorescence samples accumulated more m/z 209.1537 and 825.4698, putatively identified as Undecanoic acid and Arabidopside B, respectively (Table 2). Ws-3 inflorescences accumulated more m/z 233.1163, 233.1694, and 249.1849, putatively identified as (+)-Epijasmonic acid, Lauric acid, and Myristoleic acid, respectively (Table 2).

#### Class 5: alkaloids

Only three metabolites with significant differences were found that belong to this class. Col-0 leaves accumulated more m/z 190.0039, putatively identified as Quinolic acid, whereas Ws-3 accumulated more m/z 199.0751, putatively identified as N-hydroxy tryptamine. The m/z 363.0392 was distinct in inflorescence samples and showed higher accumulation in Ws-3 than in the Col-0 accession.

#### Class 6: glucosinolates biosynthesis and degradation

Three metabolites found in leaf and in inflorescence samples, respectively, belong to class 6.

We detected one distinct metabolite in leaf and one in inflorescence samples: m/z 235.0595, putatively identified as 2-(4′-Methylthio)butylmalic acid (Table 1), and m/z 465.0834, putatively identified as 6-Methylsulfinylhexyl glucosinolate, respectively (Table 2). Moreover, we could distinguish that Col-0 accumulated more m/z 223.0572 and 256.1438 in leaf samples (Table 1), and more m/z 223.0570 and 256.1437 in inflorescence samples (Table 2).

#### Class 7: amino acids and amino acid metabolism

Class 7 contains metabolites involved in the biosynthesis or metabolism of amino acids. Four and eight metabolites that belong to this class were identified in leaf and inflorescence samples, respectively, and only two of them were detected in both tissues.

Ws-3 leaves accumulated more of the following m/z: 221.0315, 244.0489, 275.1138, and 313.0989 (Table 1), whereas Ws-3 inflorescences accumulated more of the m/z 188.0738, 199.0751, 213.0917, 221.0315, 244.0489, 291.1242, and 385.1300 (Table 2) than the respective Col-0 tissues. On the other hand, Col-0 inflorescence samples accumulated more of m/z 231.0831 (Table 2) than Ws-3 inflorescence.

#### Class 8: carbohydrates

We putatively identified two metabolites that belong to this class and showed differential behavior among accessions. One of them was Sucrose (m/z 343.1212) that accumulated more in Col-0 than in Ws-3 leaves, but was more abundant in Ws-3 than in Col-0 inflorescences (Table 1). The second one was D-Ribulose 1,5-bisphosphate (m/z 328.9407), which was more abundant in Col-0 inflorescences samples.

#### Class 9: vitamins

We found significant variation in one metabolite that belongs to this class, m/z 250.0505, putatively identified as [5-Hydroxy-4-(hydroxymethyl)-6-methyl-3-pyridinyl]methyl dihydrogen phosphate, which was more abundant in Ws-3 (Table 1) than Col-0 leaves.

**Figure 4:**
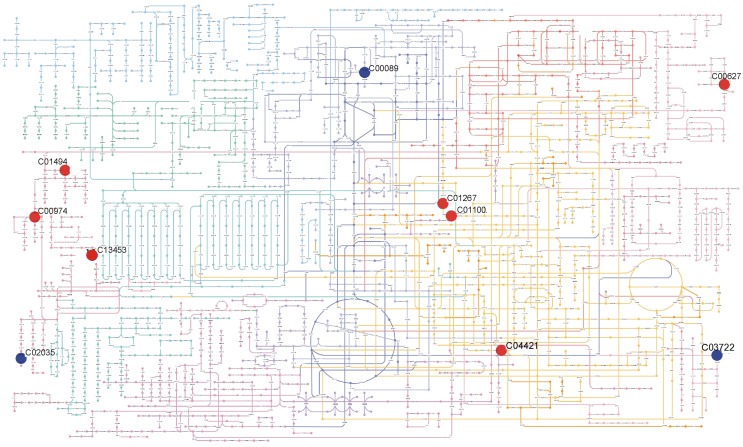
**Summary of metabolic differences detected in leaves of Col-0 and Ws-3 *Arabidopsis thaliana* accessions**. The figure shows the metabolites present in KEGG Atlas with its KEGG ID: Ferulic acid (C01494); (+)-Dihydrokaempferol (C00974); N-Succinyl-LL-2,6-diaminoheptanedioate (C04421); [5-Hydroxy-4-(hydroxymethyl)-6-methyl-3-pyridinyl]methyl dihydrogen phosphate (C00627); 3-(1H-Imidazol-4-yl)-2-oxopropyl dihydrogen phosphate (C01267); L-Histidinol phosphate (C01100); 5-(4-Hydroxy-2,2,6-trimethyl-7-oxabicyclo[4.1.0]hept-1-yl)-3-methyl-2,4-pentadienal (C13453); Quinolinic acid (C03722); Sucrose (C00089); Gibberellin A20 (C02035). Positioning of metabolites on the metabolic map was made using Pathway Projector (Kono et al., 2009). Red circles indicate metabolites that were significantly (*p* ≤ 0.01) more abundant in Ws-3, while blue circles indicate metabolites that were significantly (*p* ≤ 0.01) less abundant in Ws-3.

**Figure 5:**
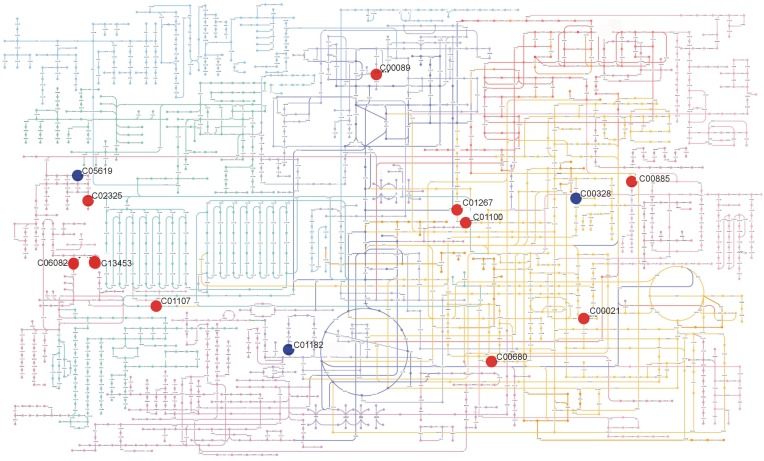
**Summary of metabolic differences detected in inflorescences of Col-0 and Ws-3 *Arabidopsis thaliana* accessions**. The figure shows the metabolites present in KEGG Atlas with its KEGG ID: Sucrose (C00089); Sinapyl alcohol (C02325); (s)-(+)-Abscisic acid (C06082); 5-(4-Hydroxy-2,2,6-trimethyl-7-oxabicyclo[4.1.0]hept-1-yl)-3-methyl-2,4-pentadienal (C13453); Mevalonate 5-phosphate (C01107); Meso-2,6-Diaminoheptanedioate (C00680); Imidazole acetol phosphate (C01267); L-Histidinol phosphate (C01100); 2-Amino-4-({[5-(6-amino-9H-purin-9-yl)-3,4-dihydroxytetrahydro-2-furanyl]methyl}sulfanyl)butanoic acid (C00021); Isochorismate (C00885); L-Kynurenine (C00328); D-Ribulose 1,5-bisphosphate (C01182); 5-Hydroxyferulic acid (C05619). Positioning of metabolites on the metabolic map was made using Pathway Projector (Kono et al., 2009). Red circles indicate metabolites that were significantly (*p* ≤ 0.01) more abundant in Ws-3, while blue circles indicate metabolites that were significantly (*p* ≤ 0.01) less abundant in Ws-3.

In summary, metabolic profiling revealed distinct metabolic phenotypes for each accession and tissue. The metabolic phenotype included metabolites from at least 9 different classes. Figures 4, 5, as an example, represent some of the metabolic differences observed in leaf and inflorescence samples. Besides the potential use of these metabolites for identification of accession and tissues, some of them like sucrose, gibberellins A20, D-Ribulose 1,5-biphosphate, are interesting for further studies that could help to understand the morphological differences as well as the growth potential of the accessions.

## Discussion

### Distinct metabolic phenotypes were detected for different accessions and tissues

Different metabolomic approaches have been undertaken seeking to establish a link between genotypes and phenotypes, e.g., some studies have described the variation of individual and specific classes of metabolites and the genetics involved in their control (Kliebenstein et al., 2001b; Wentzell et al., 2007; Chan et al., 2009), while others have described the natural variation of the metabolome of Arabidopsis through untargeted metabolomics analysis (Keurentjes et al., 2006; Rowe et al., 2008; Joseph et al., 2013, 2014).

Those studies uncovered qualitative and quantitative variation in metabolite accumulation between Arabidopsis accessions. The combination of metabolic profiling and genetics allowed the identification of QTLs associated for about 75% of the mass signals (Keurentjes et al., 2006). Other studies showed that metabolite QTLs were organized in 6 clusters with phenotypic effects (Rowe et al., 2008; Fu et al., 2009). Some of these clusters were associated with central metabolism (Rowe et al., 2008) and others to previously identified secondary metabolite loci (Kliebenstein et al., 2001a,b; Hansen et al., 2007; Wentzell et al., 2007). Furthermore, there is evidence that different factors like environment, tissue type, and plant age affect the outcomes of the genetic network controlling metabolism in Arabidopsis (Wentzell et al., 2008; Wentzell and Kliebenstein, 2008).

In our study we observed natural metabolic variation among two different tissues of the accessions Col-0 and Ws-3, which are commonly used in research. The mass profiles of each tissue of the different accessions presented quantitative and qualitative variation, allowing us to distinguish among these accessions and tissues in terms of their metabolic profiles.

We detected more than 14,000 and 17,000 peaks from inflorescences and leaves, respectively, in the two accessions. 222 high quality features presented significant differences (*p* ≤ 0.01) in leaf samples and 418 high quality features did in inflorescence samples (*p* ≤ 0.01). From these metabolites that presented significant differences we could putatively identify 26 and 36 metabolites in leaf and in inflorescence samples (Tables 1, 2), with 17 of those metabolites present in both tissues in the two accessions (Supplementary Table S1). Although many signals remain unidentified, we created a Random Forest Model, which permits the classification of both tissue and accession based on their metabolic fingerprint. The model is predictive and may be employed for the correct identification of otherwise indistinguishable plants.

### Nine metabolite classes were differentially accumulated in different accessions and tissues

In this study we found quantitative variation in nine metabolite classes, indicating different metabolite compositions in each accession and tissue.

Several studies using *A. thaliana* natural accessions have shown that differential gene expression exists among the accessions. The most differentially expressed genes concerned to the response to the biotic environment, including pathogen defense and the production of glucosinolates (West et al., 2006, 2007; Kliebenstein et al., 2006a,b; van Leeuwen et al., 2007; Gan et al., 2011). In agreement with these observations, our study showed quantitative and qualitative variation in metabolites related to pathogen defense.

The glucosinolates are a group of naturally occurring metabolites in the Brassicales order involved in plant defense (Wittstock and Burow, 2010). These thioglucosides are derived from a variety of protein amino acids (Met, Leu, iso-Leu, Val, Trp, and Phe) (Kliebenstein et al., 2001b; Keurentjes et al., 2006).

Particularly, in our extraction conditions, we observed one distinct metabolite for leaves (m/z 235.0595; Table 1) and one for inflorescences (m/z 465.0834; Table 2), and a different accumulation pattern for certain metabolites in different accessions: m/z 223.0572 and 256.1438 (Table 1) were more abundant in Col-0 leaf samples, and m/z 223.0570 and 256.1437 (Table 2) in Col-0 inflorescences. These metabolites were putatively identified to participate in glucosinolate biosynthesis and degradation.

Other studies reported glucosinolate variation in leaves and seeds of Arabidopsis accessions (Kliebenstein et al., 2001b; Matsuda et al., 2010), and they have been used to discriminate among some *A. petrea* populations (Davey et al., 2008). They also have been shown to be subjected to genetic variation in Arabidopsis (Kliebenstein et al., 2001b).

Other metabolites, classified in our study as belonging to class 4, were differentially accumulated among tissues and accessions. Ws-3 inflorescences accumulated more (+)-Epijasmonic acid (m/z 233.1163; Table 2) than Col-0, while Col-0 inflorescences more Arabidopside B (m/z 825.4698; Table 2). Jasmonic acid and Methyl jasmonate play an essential role in plant defense responses, pollen development and leaf growth control by repressing cell proliferation (Świątek et al., 2004; Zhang and Turner, 2008; Chen et al., 2013; Noir et al., 2013). Arabidopside B seems to have an inhibitory effect on root growth and a possible role as a reservoir for slow release of free OPDA, a Jasmonate precursor (Kourtchenko et al., 2007).

Also, it was interesting to note the differential accumulation of Lauric acid (m/z 223.1696) in the leaves of the two accessions, being Col-0 the one that accumulated the most in leaves and Ws-3 in inflorescences. It has been demonstrated that Lauric acid can be elongated and desaturated into Linolenic acid that then is incorporated into Jasmonic acid and Methyljasmonate (Afitlhile et al., 2004).

Phenylpropanoid pathway metabolites are also known for their protective roles (Buer et al., 2010; Fraser and Chapple, 2011). We observed a distinct pattern of accumulation of metabolites belonging to the flavonoid branch of this pathway among tissues and accessions, inflorescences being the samples that presented the most diversity in these compounds. Many of these compounds have also been considered as chemical messengers, physiological regulators and cell cycle inhibitors (Buer et al., 2010; Falcone Ferreyra et al., 2012). Furthermore, a distinct pattern of accumulation of lignin precursors was also identified here as well as differences in the content of Sinapate esters.

The rate of plant growth depends on a combination of photosynthetic carbon (C) assimilation rate and developmental programs that determine how rapidly metabolites are used for growth, although the molecular and genetic basis are not well-understood (Sulpice et al., 2009). It has been reported that natural variation in the level of central metabolites exists (Loudet et al., 2003; Calenge et al., 2006) and that there are positive and negative correlations between these metabolites and biomass (Meyer et al., 2007; Sulpice et al., 2010). Metabolites that are negatively correlated with biomass were sucrose, glucose- and fructose-6-phosphate, which link carbon flow from photosynthesis and starch and sucrose metabolism with cell wall formation, the TCA cycle members citric acid, succinate or malic acid, as well as the amino acids glutamine and phenylalanine (Meyer et al., 2007). In our study, we observed differences in sucrose content in leaf and inflorescence samples between accessions, and of D-ribulose 1,5 biphospate in inflorescence samples as well as differences in the content of phenylalanine and phenylalanine derived compounds that contribute to cell wall formation (ferulic acid, sinapine). Houshyani et al. (2012) observed significant differences among Arabidopsis accessions for some primary metabolites, e.g., fructose, 1-methyl-alpha-D-glucopyranoside, glucopyranose, sucrose, and L-glutamic acid. Metabolite QTLs were also associated with central metabolism, suggesting that differences in central carbon metabolism can exist among accessions (Rowe et al., 2008; Houshyani et al., 2012).

Plant hormones affect gene expression and transcription levels promoting cellular division, growth and differentiation, and directing developmental programs that determine how rapidly metabolites are used for growth (Alabadi et al., 2009; Sulpice et al., 2010).

Paparelli et al. (2013) showed that plant size is determined by a mechanism in which carbohydrates produced by photosynthesis modulate the synthesis of gibberellins (Paparelli et al., 2013). In our study, we observed a different pattern of GAs and sucrose accumulation among accessions (Tables 1, 2 and Figures 4, 5), which could be further investigated to establish a correlation with the growth phenotypes.

Moreover, differences between accessions in the content of abscisic acid or intermediates in abscisic acid biosynthesis were found. Abscisic acid is well-known for its effect on seed germination, flowering, and during plant response to environmental stress and plant pathogens. Col-0 and Ws-3 present differences in flowering time that could eventually be explained by the differences here found in the content of GAs and ABA, hormones that concur to regulate this process. Recent studies showed that ABA potentially delays flowering under unstressed conditions, but promotes it when plants are stressed (Finkelstein, 2013).

Further work must be done to investigate whether there is a correlation between the hormonal differences and the metabolic signatures found in each accession that could explain the morphological differences among accessions.

## Concluding remarks

In this work we found a distinct metabolic phenotype of each tissue and Arabidopsis accession studied. We found quantitative variation in nine metabolite classes, resulting in different compositions of metabolites in each accession and tissue. Moreover, a predictive Random Forest Model was made that is able to reliably classify tissue type and accession of samples based on LC-MS profiles.

The metabolite signature of accessions found in this work could set a basis for future studies to understand how these profiles correlate with their respective phenotype. For example, by exploring its correlation with interesting developmental processes, like cell division, cell expansion, flowering time, and biomass production. Moreover, knowledge of metabolite natural diversity could help to direct plant breeding approaches.

## Author contributions

MS performed the sample preparations and AC performed chromatography and mass spectrometry experiments. MS, NM, RW, and SF conceived the project, designed the experiments, and analyzed the data. RW performed data processing, Random Forest Model development, and statistical analysis. MS, NM, RW, and SF drafted the manuscript. All authors read and approved the final manuscript.

### Conflict of interest statement

The authors declare that the research was conducted in the absence of any commercial or financial relationships that could be construed as a potential conflict of interest.
