# Supplementary material for: Metabolic fingerprinting of Arabidopsis thaliana accessions
Source: Front Plant Sci. 2015 May 27;6:365. doi: 10.3389/fpls.2015.00365 (PMC4444734; doi:10.3389/fpls.2015.00365)
Supplement: Supplementary file 1 [file Table1.PDF]

Supplemental Table S1. Putatively identified metabolites found in common in leaves and inflorescences.

Class	m/z	Ionization mode	Name	Chem Spider ID	Fold change (Ws-3/Col-0)	p-value
1	195.0648	[M+H]+	Ferulic acid	393368	2.4	7.85E-003
1	197.0803	[M+H]+	5-Hydroxyconiferyl alcohol	4445309	1.4	2.52E-002
1	211.0571	[M+H]+	5-Hydroxyferulic acid	141117	0.9	1.37E-001
1	219.0626	[M+Na]+	5-Hydroxyconiferyl alcohol	4445309	1.0	8.72E-001
1	311.1692	[M+H]+	Sinapine	80576	0.7	2.93E-005
2	249.1464	[M+H]+	Abscisic acid aldehyde	14797236	1.4	1.82E-001
2	273.1457	[M+Na]+	5-(4-Hydroxy-2,2,6-trimethyl-7-oxabicyclo[4.1.0]hept-1-yl)-3-methyl-2,4-pentadienal	21172770	1.4	1.79E-003
2	333.1742	[M+H]+	Gibberellin A4	10222155	2.0	1.36E-002
2	343.2645	[M+Na]+	1,22-Docosane diol	190585	1.5	1.79E-005
2	355.1444	[M+Na]+	Gibberellin A51	391672	1.4	3.40E-002
2	355.1579	[M+Na]+	gibberellin A20	20015789	0.6	3.72E-003
2	369.1222	[M+Na]+	Gibberellic acid	6223	1.3	6.84E-003
2	387.1347	[M+Na]+	Gibberellin A8	4444216	0.9	3.51E-001
3	323.0307	[M+H]+	Cyanidin 3-O-[2''-O-(2'''-O-(sinapoyl) xylosyl) glucoside] 5-O-glucoside	61546	3.3	2.54E-005
3	329.0675	[M+Na]+	Leucocyanidin	64694	1.5	8.34E-003
4	223.1695	[M+Na]+	Lauric acid	3756	0.4	4.25E-004
4	825.4701	[M+Na]+	Arabidopside B	10192487	1.1	5.68E-001
5	190.0039	[M+Na]+	Quinolinic acid	1037	0.6	2.57E-006
6	223.0572	[M+Na]+	5-Methylsufinylpentyl nitrile	1363309	0.8	5.64E-002
6	256.1438	[M+Na]+	Hexahomomethionine	21865788	0.7	1.22E-003
7	221.0315	[M+H]+	3-(1H-Imidazol-4-yl)-2-oxopropyl dihydrogen phosphate	770	1.1	1.94E-003
7	244.0489	[M+Na]+	L-Histidinol phosphate	388515	1.2	6.71E-003
